# A novel classification system for avulsion fractures of the distal fibula based on arthroscopy-assisted measurement and significance to surgery and rehabilitation

**DOI:** 10.3389/fmed.2026.1921307

**Published:** 2026-09-10

**Authors:** Linguo He, Qiang Teng, Wenrui Wu, Fangling Wang, Boyuan Zheng, Dahai Hu, Xiaofei Zheng, Huige Hou

**Affiliations:** 1 Department of Orthopedics, Dongsheng Hospital of Zhongshan, Zhongshan, Guangdong, China; 2 Department of Sports Medicine, The First Affiliated Hospital, Guangdong Provincial Key Laboratory of Speed Capability, The Guangzhou Key Laboratory of Precision Orthopedics and Regenerative Medicine, Jinan University, Guangzhou, China

**Keywords:** arthroscopy, avulsion fracture, classification, fibula, rehabilitation

## Abstract

**Background:**

There are difficulties in clinically diagnosing avulsion fractures of the distal fibula, although arthroscopic examination is an effective diagnostic method. To propose a novel classification scheme for avulsion fractures of the distal fibula, and further provide guidance for surgical and rehabilitation strategies.

**Methods:**

Data from 152 patients diagnosed with avulsion fractures of the distal fibula between February 1, 2019, and April 30, 2023, were included in this study. The characteristics of avulsion fractures of the distal fibula according to arthroscopic observations were identified, and concomitant intra-articular pathology was described and recorded.

**Results:**

Forty-five females and 107 males were enrolled. According to the size and relationship between the bone fragments and the ligaments, avulsion fractures were classified into the following types: A1 (*n* = 9 [6.0%]); A2 (*n* = 31 [20.3%]); B1 (*n* = 62 [40.8%]); B2 (*n* = 33 [21.7%]); C1 (*n* = 13 [8.6%]); and C2 (*n* = 4 [2.6%]). To further accurately locate avulsion fractures, fracture position was divided into anterior, junctional, and posterior areas. Most avulsion fractures occurred in the anterior and junctional areas (82.9%). Additionally, there was an association between the incidence of pathological soft tissue occupying the anterior compartment (*p* < 0.001), osteochondral defect (*p* = 0.035), and the type of avulsion fracture.

**Conclusion:**

This study established a new classification system for avulsion fractures of the distal fibula. Based on this classification system, we have proposed a serial management for different types of avulsion fractures, which provides certain reference value for clinical treatment.

## Introduction

Avulsion fracture(s) of the distal fibula, a commonly occurred sports injury, locates at the insertion of anterior talofibular ligament (ATFL) or calcaneofibular ligament (CFL) (1–5). Ankle sprain is an important inducing factor (6). In addition, due to the unique location of the fracture, it is easy to misdiagnose using standard anteroposterior or lateral plain X-ray films (6–8). Avulsion fractures of the distal fibula are often accompanied by damage to the surrounding ligaments (9–11). If avulsion fractures of the distal fibula are poorly managed or missed, chronic ankle instability usually emerges. Consequently, appropriate diagnosis and treatment are of vital importance for this condition.

Presently, avulsion fractures are mainly classified as a type of fracture that is clinically insignificant and has a good prognosis (1, 12, 13). Treatment strategies for avulsion fractures of the distal fibula are divided into conservative and surgical. Conservative treatment includes pain relief and physical rehabilitation. In case conservative treatment fails, or incomplete fragments or subfibular ossicles induces pain and instability (10, 14–18), surgical treatment is suggested. Surgical treatment includes the removal of small bone fragments, or the reduction of bone fragments followed by fixation and repair of ligament insertion points. Although there are various methods to treat avulsion fractures of the distal fibula, the position, size, and relationship between the bone fragments and ligaments are rarely considered. Therefore, it is necessary to classify avulsion fractures of the distal fibula based on these factors and adopt corresponding treatment strategies.

The purpose of this study is to propose a new classification scheme for avulsion fractures of the distal fibula based on arthroscopic observation of the position, size, and relationship between the bone fragments and ligaments, to provide a reference for the selection of appropriate treatment strategies.

## Materials and methods

This research was approved by the Ethics Committee of the local hospital. Data of patients diagnosed with avulsion fractures of the distal fibula, between February 1, 2019, and April 30, 2023, were included in this study. All procedures were performed by the identical experienced surgeon. Each included case had preoperative X-ray, computed tomography or magnetic resonance imaging of the index joint. Basic characteristics were also recorded.

### Screening criteria

The inclusion criteria were as follows: (1) avulsion fracture(s) of the distal fibula confirmed by physical and imaging evidence; (2) failure of conservative treatment for 2 weeks, or accompanied by ankle ligament injury and ankle instability; (3) and no previous surgery or trauma of the index limb. Individuals with multiple ankle fractures in the medial and posterior malleolus, those with open fracture(s), definite bone malformations, such as flat foot, high arch deformity, talocalcaneal bridge, systemic multi-ligament relaxation, ankle joint fibrosis, occupation of the lateral sulci, and other diseases, and those with incomplete data were excluded.

### Arthroscopic measurement

Anteromedial, anterolateral and accessory anterolateral portals were established. The anteromedial portal is located at the level of the ankle line and close to the medial margin of the anterior tibial muscle tendon. Anterolateral portal is located 0.5 cm distal to the ankle line and near the lateral side of the third peroneal muscle tendon. Accessory anterolateral portal is located 0.5 cm at the distal end of the ankle line, close to the anterior side of the fibula and 1.0 cm from the fibular tip.

At the medial joint line of the tibialis anterior muscle in front of the ankle joint, a needle was inserted into the joint capsule and 15–20 mL of normal saline was injected. Incision was then established. Subcutaneous tissue was separated to introduced the arthroscope (Arthrex, 28731 BWA, 4.0 mm) through anteromedial portal. Under arthroscopic monitoring, a guide-needle was positioned at the third peroneal muscle and a lateral incision was made to construct an anterolateral portal. Subsequently, the distal end of the fibula, joint cavity, ATFL, and CFL were observed under arthroscopy.

**Figure 1:**
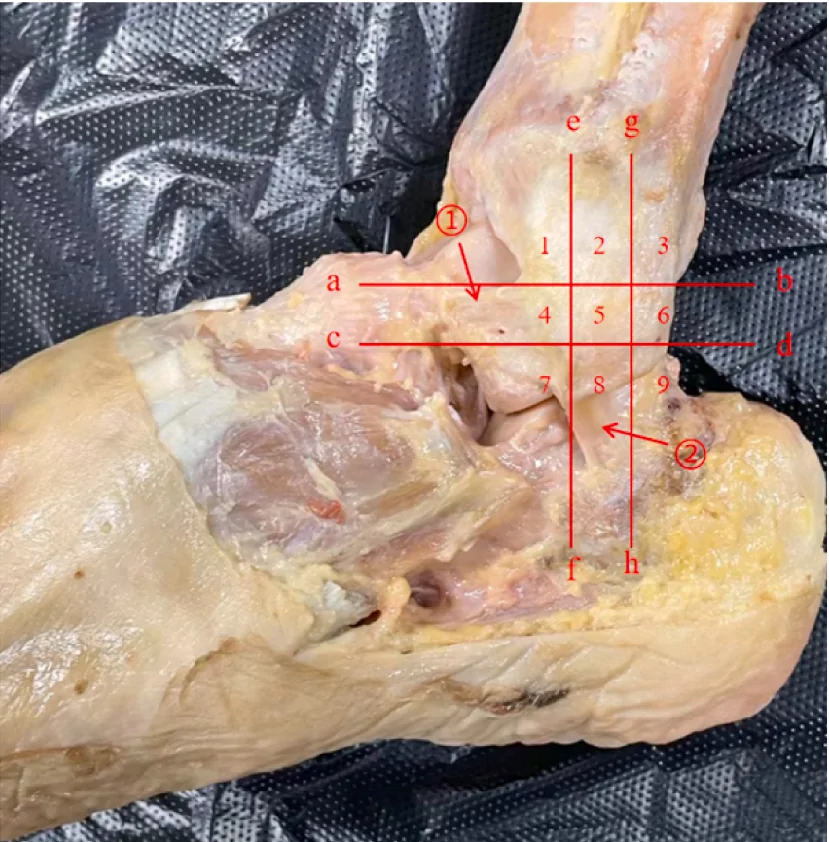
The schematic diagram shows nine areas of the fibula viewed from the side. Region 1, 4, and 7 was defined as the anterior area. Region 2, 5, and 8 was defined as the junctional area. Region 3, 6, and 9 was defined as the posterior area. ① Anterior talofibular ligament; ② calcaneofibular ligament.

Arthroscopic records were reviewed by one experienced surgeon and verified by another experienced surgeon. Characteristics of different types of avulsion fracture(s) of the distal fibula were identified under the microscope. Fragment size (i.e., the length of the longest edge of the bone fragment) was assessed using preoperative CT, and fragment location was observed under arthroscopy. According to position, size, and relationship between the fracture block and the ligaments under arthroscopy, avulsion fractures of the distal fibula were classified arthroscopically in to steps. First, the fracture was categorized according to size: A (< 5 mm), B (≥ 5 mm, and ≤ 10 mm), and C (> 10 mm). Next, based on whether the bone fragment is separated from the ligament, three conditions were proposed: type 1 (No) and type 2 (Yes). Finally, based on the location of the avulsed bone block at the distal end of the fibula, it was divided into three zones: 1 (anterior area); 2 (junctional area); and 3 (posterior area) (Figure 1). Concomitant intra-articular pathology was also documented.

For cases where the bone fragment is located at the junction of two zones, we adopt the principle of ‘dominant location’. If the center of the fracture fragment was located in the overlapping boundary area, the fragment was assigned to the zone where the majority (> 50%) of its volume or attachment site was situated. For bone fragments larger than 10 mm, they are classified only as types C1 and C2 based on their relationship with the ligament.

### Statistical analysis

Prism software (GraphPad Inc.) and SPSS v19.0 (IBM Corporation) were used for data analysis (19). The relationship between the avulsion fractures of the distal fibula type and intra-articular pathology was tested using the Kruskal–Wallis H test. The Kappa value was used to assess the consistency between the two observers’ findings. Measurement data are expressed as mean ± standard deviation (SD). Differences with *p* < 0.05 were considered to be statistically significant.

## Results

One hundred sixty-six patients were screened. According to the inclusion and exclusion criteria, 14 were excluded: previous foot and ankle surgery or fractures (*n* = 7); multiple ankle fractures (*n* = 3); incomplete data (*n* = 2); and high arch deformity (*n* = 2). Ultimately, 152 cases were analyzed.

Among the included patients, 45 (29.6%) were female and 107 (70.4%) were male. The mean age was 29.1 ± 6.8 years. The left ankle was affected in 63 patients (41.4%) and the right in 89 (58.6%).

Through arthroscopic observation, this study found some conformant pathologies in addition to avulsion fracture(s) of the distal fibula, including loose body (*n* = 9 [5.9%]), peroneal tendon complaints (*n* = 7 [4.6%]), pathological soft-tissue occupying the anterior compartment (*n* = 103 [67.8%]), osteoarthritis (*n* = 2 [1.3%]), osteochondral defect (*n* = 13 [8.6%]), and cartilage lesions (*n* = 3 [2.0%]) (Table 1). In addition, a significant association was noted between the incidence of pathological soft tissue occupying the anterior compartment (*p* < 0.001), osteochondral defect (*p* = 0.035), and type of avulsion fracture (Table 1).

**Table 1:** Ankle pathology observed under arthroscopy.

Concomitant pathology	*p-*value	Type of the avulsion fracture at the distal end of the fibula														Overall (*n* = 152)
		Type A (*n* = 40, 26.3%)						Type B (*n* = 95, 62.5%)						Type C (*n* = 17, 11.2%)		
		A1.1 (*n* = 0)	A1.2 (*n* = 0)	A1.3 (*n* = 9)	A2.1 (*n* = 8)	A2.2 (*n* = 23)	A2.3 (*n* = 0)	B1.1 (*n* = 22)	B1.2 (*n* = 40)	B1.3 (*n* = 0)	B2.1 (*n* = 24)	B2.2 (*n* = 9)	B2.3 (*n* = 0)	C1 (*n* = 13)	C2 (*n* = 4)	
Loose body	0.238	0	0	0	1 (12.5%)	2 (8.7%)	0	1 (4.5%)	1 (2.5%)	0	1 (4.2%)	0	0	2 (15.4%)	1 (25.0)	9 (5.9%)
Peroneal tendons complaint	0.306	0	0	0	0	1 (4.3%)	0	0	2 (5.0%)	0	1 (4.2%)	1 (11.1%)	0	1 (7.7%)	1 (25.0%)	7 (4.6%)
Pathological soft-tissue occupying the anterior compartment	<0.001	0	0	4 (44.4%)	5 (62.5%)	17 (73.9%)	0	17 (77.3%)	23 (57.5%)	0	19 (79.2%)	5 (55.6%)	0	11 (84.6%)	2 (50.0%)	103 (67.8%)
Osteoarthritis	n.s.	0	0	0	0	0	0	0	0	0	0	1 (11.1%)	0	1 (7.7%)	0	2 (1.3%)
Osteochondral defect	0.035	0	0	1 (11.1%)	0	2 (8.7%)	0	0	4 (10.0%)	0	3 (12.5%)	1 (11.1%)	0	2 (15.4%)	0	13 (8.6%)
Cartilage lesions	n.s.	0	0	0	0	1 (4.3%)	0	0	1 (2.5%)	0	0	0	0	1 (7.7%)	0	3 (2.0%)
No intra-articular injury	<0.001	0	0	6 (66.7%)	4 (50.0%)	10 (43.5%)	0	8 (36.4%)	5 (12.5%)	0	1 (4.2%)	0	0	0	0	34 (22.4%)

n.s., non-significant.

**Figure 2:**
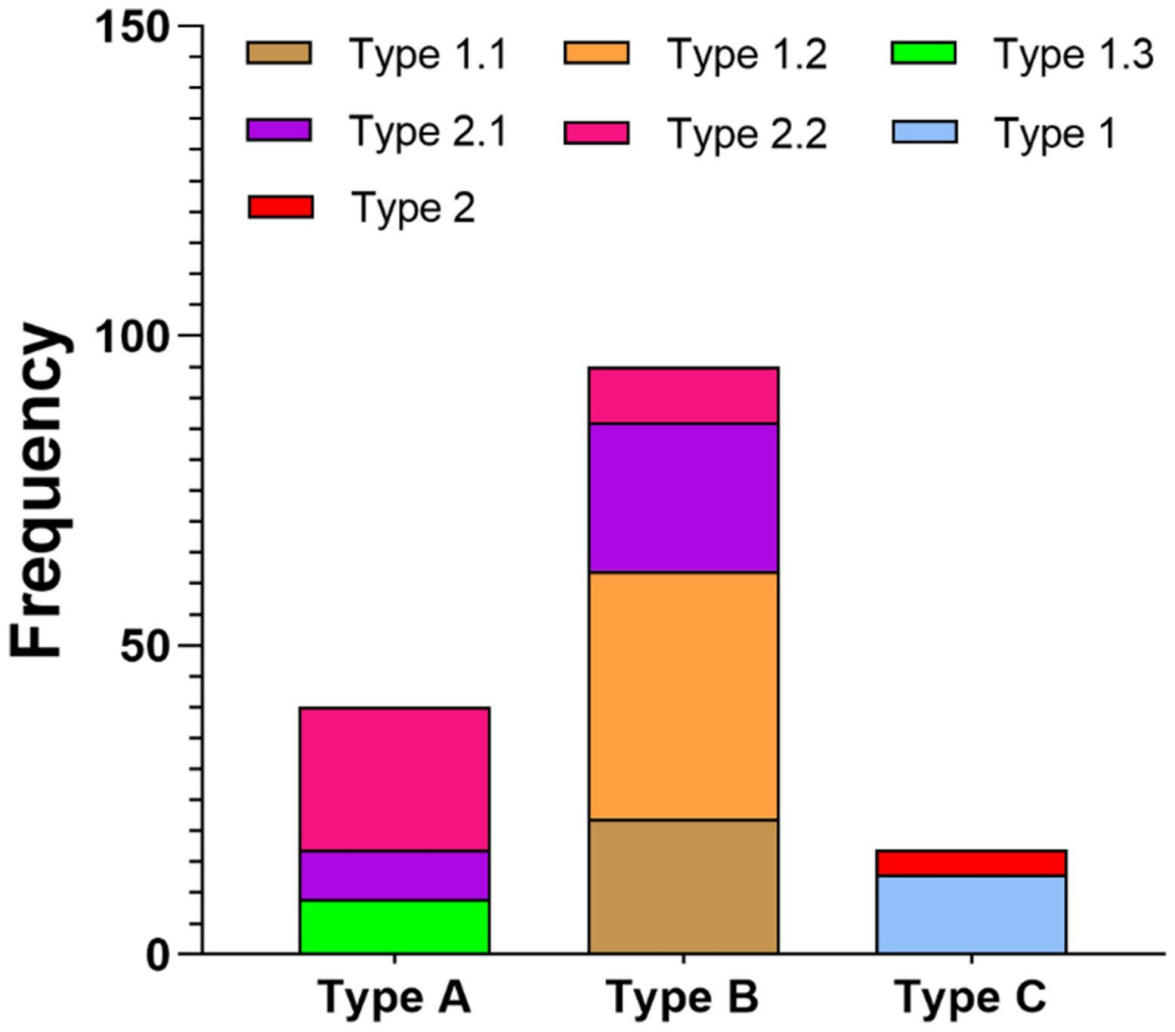
The histogram figure for the number of patients included in different types of the avulsion fracture.

As showed in Figure 1, the distal end of the fibula was divided into nine regions (9). According to the size of the fracture block, avulsion fracture(s) of the distal fibula were classified as follows: type A (*n* = 40 [26.3%]); type B (*n* = 95 [62.5%]); and type C (*n* = 17 [11.2%]) (Table 1 and Figure 2). Arthroscopic analysis revealed that that when the size of the fracture block was < 5 mm, it was usually located in region 4 or region 8, often affecting the ATFL or CFL, or were not impacted. When the size of the fracture block was > 10 mm, it had a significant impact on the ATFL or CFL.

**Figure 3:**
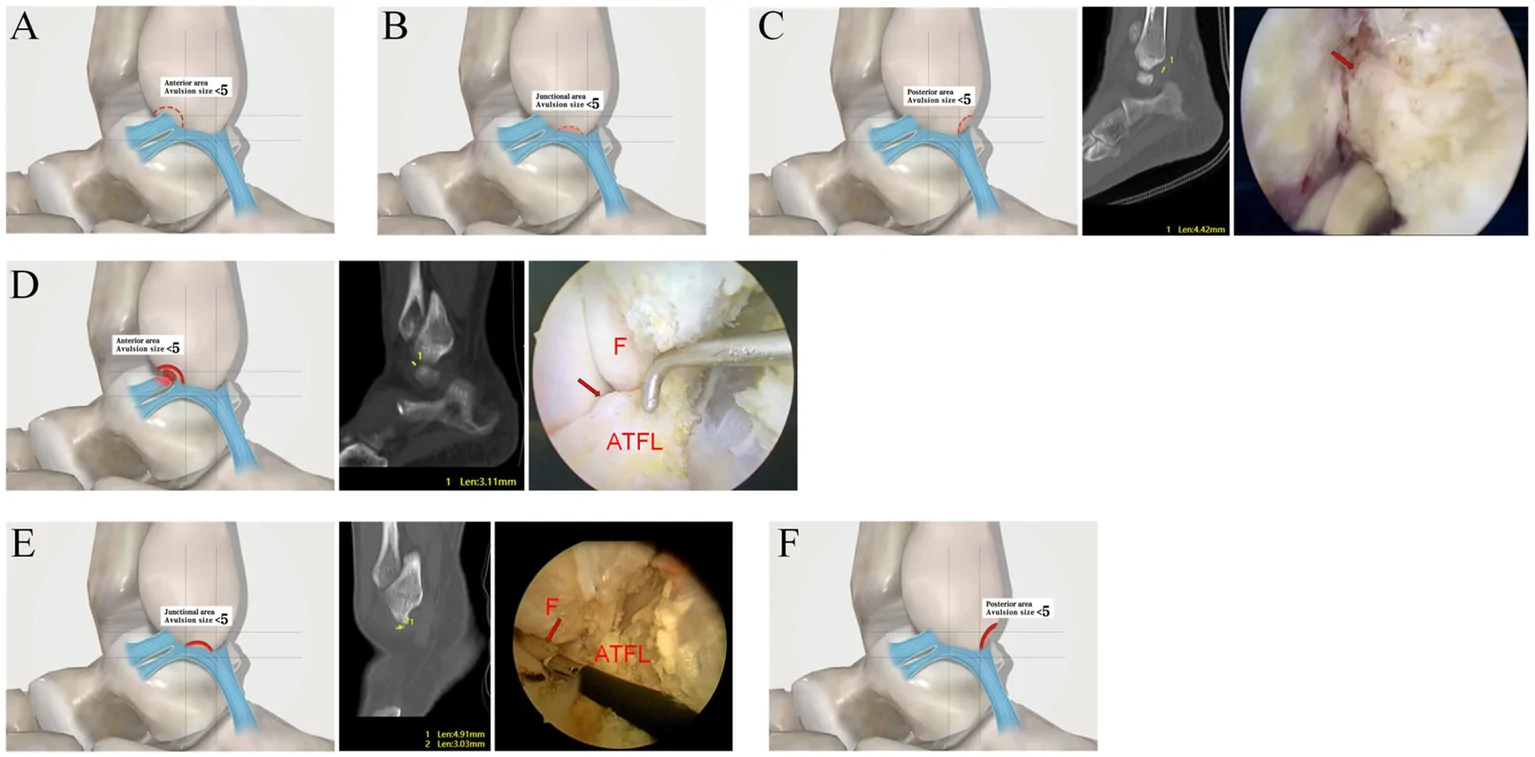
The schematic diagram, preoperative CT, and arthroscopic images of the type A avulsion fracture of the distal fibula. **(A)** Type A1.1, **(B)** Type A1.2, **(C)** Type A1.3, **(D)** Type A2.1, **(E)** Type A2.2, **(F)** Type A2.3. The dashed line represents that the bone has not been completely separated from the ligament. The solid line represents that the bone has been completely separated from the ligament. ATFL, anterior talofibular ligament; F, fibula; the arrow indicates an avulsion fracture.

**Figure 4:**
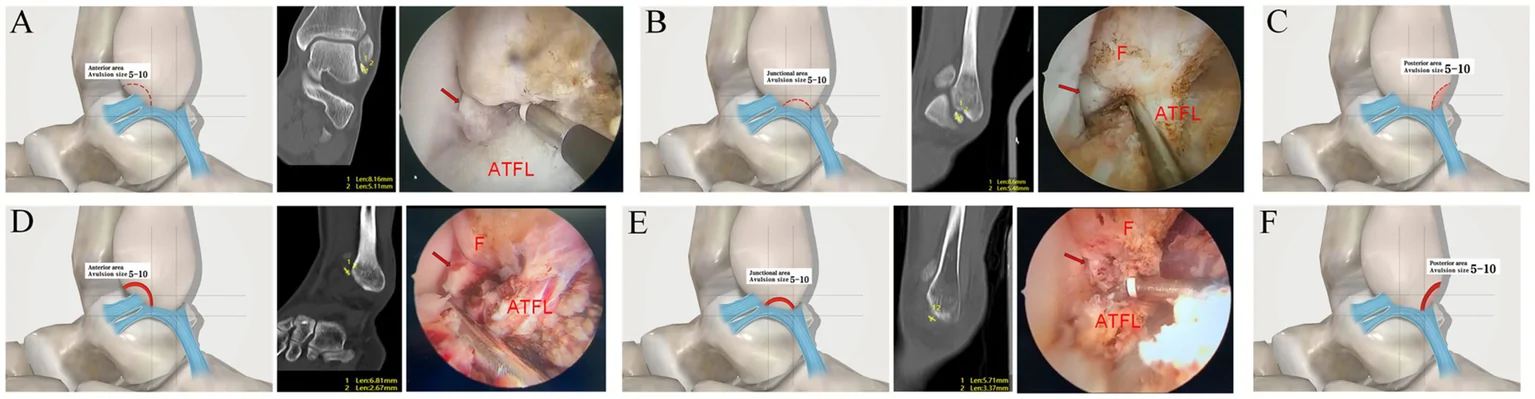
The schematic diagram, preoperative CT, and arthroscopic images of the type B avulsion fracture of the distal fibula. **(A)** Type B1.1, **(B)** Type B1.2, **(C)** Type B1.3, **(D)** Type B2.1, **(E)** Type B2.2, **(F)** Type B2.3. The dashed line represents that the bone has not been completely separated from the ligament. The solid line represents that the bone has been completely separated from the ligament. ATFL, anterior talofibular ligament, F, fibula, the arrow indicates an avulsion fracture.

**Figure 5:**
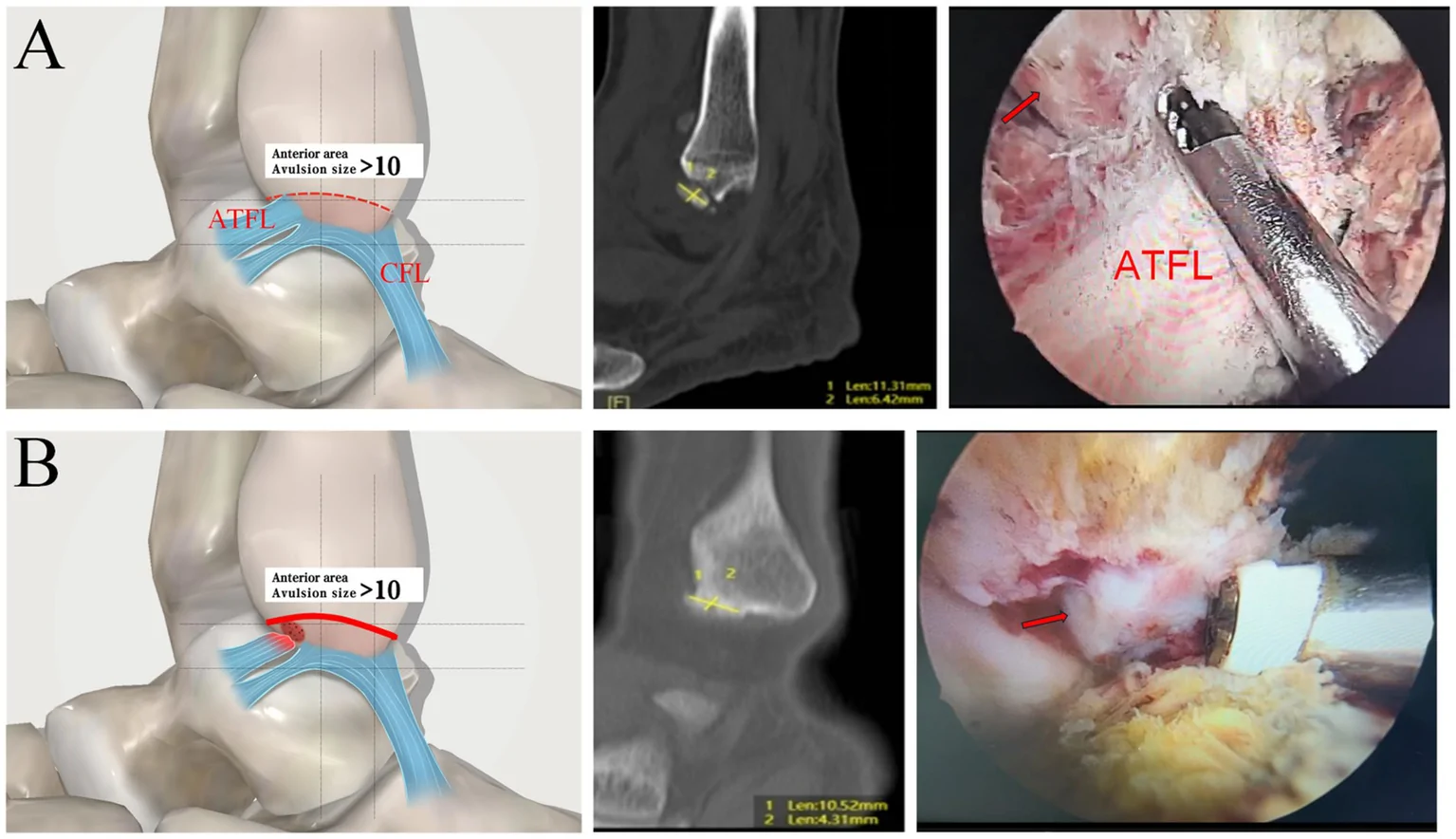
The schematic diagram, preoperative CT, and arthroscopic images of the type C avulsion fracture of the distal fibula. **(A)** Type C1, **(B)** Type C2. The dashed line represents that the bone has not been completely separated from the ligament. The solid line represents that the bone has been completely separated from the ligament. ATFL, anterior talofibular ligament; CFL, calcaneofibular ligament, the arrow indicates an avulsion fracture.

The separation of fracture blocks and ligaments often affects the stability of the joint cavity structure, leading to instability of the ankle joint. Based on whether the fracture block was separated from the ligament, it was divided it into the following 6 types: A1 (*n* = 9 [6.0%]); A2 (*n* = 31 [20.3%]); B1 (*n* = 62 [40.8%]); B2 (*n* = 33 [21.7%]); C1 (*n* = 13 [8.6%]); and C2 (*n* = 4 [2.6%]). Detailed classification of type A, type B, and type C were illustrated in Figures 3–5, respectively.

To further accurately locate avulsion fractures, the fracture position was divided into anterior, junctional, and posterior areas. As shown in Table 1, avulsion fractures of the distal fibula mainly occurred in the anterior and junctional areas (82.9%).

**Figure 6:**
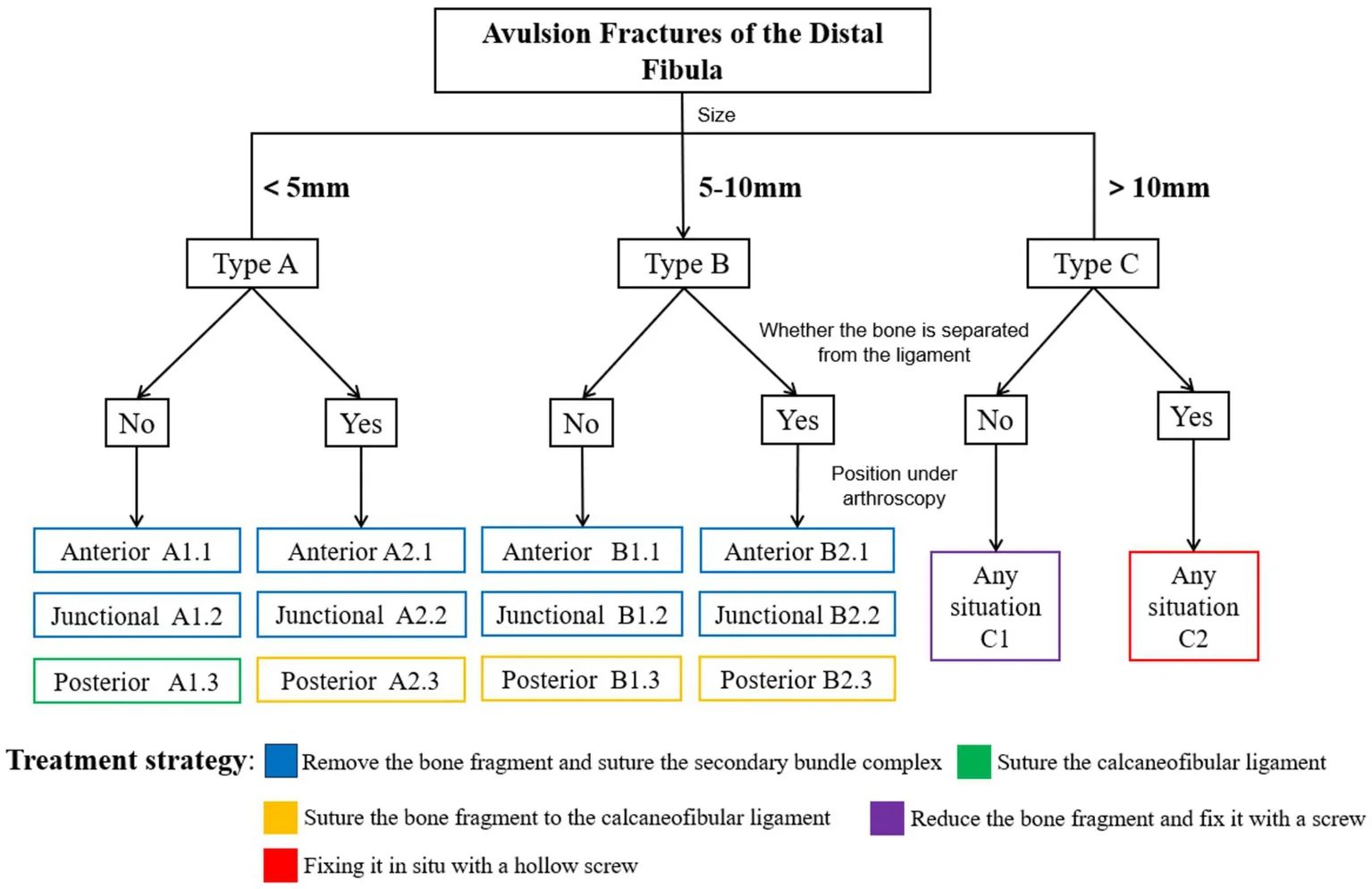
The classification criteria for avulsion fractures of the distal fibula.

Next, we propose corresponding treatment strategies for different types of avulsion fractures. As shown in Figure 6, for fracture fragments ≤ 10 mm located in the anterior or junctional zones (A1.1, A1.2, A2.1, A2.2, B1.1, B1.2, B2.1, and B2.2), we remove the fragment and repair the secondary bundle complex by suturing. For fractures located in the posterior zone with a fragment size < 5 mm (A1.3), we opt to remove the fragment and suture the CFL. For posterior zone fractures with a fragment size between 5 and 10 mm (B1.3, and B2.3), we suture the fragment together with the CFL using a lasso technique. For fractures with a fragment size > 10 mm, if the fragment is tightly connected to the ligament (C2), we perform *in situ* fixation with a hollow screw; if the fragment is loosely connected to the ligament (C1), we reduce the fragment and fix it with a screw.

## Discussion

Based on the analysis of our cohort, this study has proposed a novel classification scheme for avulsion fractures of the distal fibula based on the position, size, and relationship between the bone fragments and ligaments, and establish corresponding management measures for different types of avulsion fractures.

For avulsion fractures of the distal fibula, we often refer to the study by Zhang et al. (9), who divided it into 9 regions for positioning (9-region matrix). However, Vega et al. (20) found that the lower tract of the ATFL and the CFL had a common insertion point at the end of the fibula and were connected by arcuate fibers functioning as an independent unit (21, 22). In 2021, this result was validated in 13 freshly frozen ankle joints by Cordier et al. (23) Therefore, in this study, the 9-region matrix was simplified and divided into the anterior, junctional and posterior areas.

When the fracture block was > 10 mm, it was mainly located in regions 7–9, which significantly affects the CFL and ATFL (24). This is consistent with the observation in this study. From the results of this study, the majority of fracture block sizes were < 5 or 5–10 mm, whether this affects the ATFL, CFL or lateral ligament complex depends on the specific situation. In addition, in the study by Zhang et al., 5–10 mm fracture blocks were often located in regions 4, 7, and 8. This is consistent with the high proportion of fractures in the anterior and the junctional areas found in this study. This indicates that the classification scheme proposed in this study is feasible.

For some large-size fracture blocks, regardless of whether they affect the ATFL or CFL, we recommend surgical resection to prevent instability of the ankle joint (25, 26). However, relevant studies have shown that removing fracture fragments may cause ligament damage, thereby affecting joint stability (27, 28). Therefore, this novel classification scheme may serve as a foundation for guiding clinical practice. For different avulsion fracture classification, we can choose the best conservative or most appropriate surgical treatment strategy according to individual patient situation.

Due to the pulling effect of ligaments on the fracture block, the avulsion fracture blocks cannot stably contact the distal end of the fibula, making it difficult to complete the process of bone healing (21). In addition, improper treatment of avulsion fractures of the distal fibula may cause chronic pain and, in severe cases, develop into chronic ankle instability. Han et al. (29) removed the avulsion fracture blocks under arthroscopy for treatment. The size of the fracture bodies was as follows: < 5 mm (46%); 5–10 mm (46%); and > 10 mm (8%). Postoperative patient satisfaction reached 88%. Additionally, Diallo et al. (21) used arthroscopic assisted hollow compression screws to fix the fracture blocks (mean size, 6.3 mm), achieving good postoperative efficacy. Presently, there is no consensus regarding the treatment of avulsion fractures of the distal fibula; as such, it is of great clinical significance to establish an effective clinical classification.

Furthermore, based on the distinct types of avulsion fractures of the distal fibula identified, this study established corresponding management strategies. In a review, Schlickewei et al. (30) indicated that surgical management often leads to significant improvements in both clinical and radiographic outcomes for these fractures, while substantially reducing the incidence of chronic ankle instability. Consequently, early, precise, and effective intervention is particularly crucial for avulsion fractures of the distal fibula.

The present study had a few limitations, the first was limited sample size; as such, no corresponding patients were identified for some specific avulsion fracture(s) of the distal fibula classification. Second, the classification scheme proposed requires further verifications, especially its value in guiding the management of this condition, as well as short- and long-term prognosis. Third, this study is currently in an initial descriptive phase, and clinical validation will be required in future work. Finally, the population included in this study was predominantly composed of young and middle-aged adults, with only a limited number of adolescent participants. However, among the adolescent population, avulsion fractures of the distal fibular account for a relatively high proportion of sports-related injuries, which should not be overlooked.

## Conclusion

According to the position, size, and relationship between the bone fragments and the ligaments, this study proposed a novel classification system for the avulsion fracture of the distal fibula. In addition, the connection between ligament attachment points and avulsion fractures was also considered. The new classification scheme can provide detailed reference information for clinical treatment and facilitate the design of appropriate treatment strategies.

## Data Availability

The original contributions presented in the study are included in the article/supplementary material, further inquiries can be directed to the corresponding authors.
